# Sensory Feedback and the Dynamic Control of Movement

**DOI:** 10.1146/annurev-neuro-112723-042229

**Published:** 2025-04-08

**Authors:** Martyn Goulding, Tejapratap Bollu, Ansgar Büschges

**Affiliations:** 1 Molecular Neurobiology Laboratory, The Salk Institute for Biological Studies, La Jolla, California, USA; 2 Institute of Zoology, University of Cologne, Cologne, Germany

**Keywords:** somatosensory feedback, proprioception, motor control, spinal cord, ventral nerve cord, inhibition

## Abstract

Motor systems in animals are highly dependent on sensory information for optimal control and precision, with mechanosensory feedback from the somatosensory system playing a critical role. These mechanosensory pathways are woven into the descending feedforward pathways and local central pattern generator circuits that control and generate movement, respectively. Somatosensory feedback in mammals and insects, the two animal classes this review touches upon, is complex due to the increased demands that limbed locomotion, weight-bearing, and corrective movements place on sensorimotor control. In this review, we outline the salient features of the proprioceptive and exteroceptive sensory feedback pathways animals rely on for controlling movement and highlight some of the key principles of sensory feedback that are shared across the animal kingdom.

## INTRODUCTION

The control of movement by sensory feedback is primarily mediated by sensorimotor networks in the spinal cord of vertebrates and ventral nerve cord (VNC) of invertebrates. These local sensorimotor networks generate the patterned motor activity for all body movements, from simple reflexes to complex volitional actions. In this review, we outline some of the salient features of the proprioceptive and exteroceptive sensory feedback pathways animals rely on for motor control with a focus on mammals and insects. Somatosensory information, in addition to being used locally at the level of the spinal cord and VNC, is relayed to brain structures that exert executive control over movement via descending pathways that intersect with and engage sensorimotor circuits in the spinal cord and VNC. Interestingly, while marked differences exist in the body plans of vertebrates and invertebrates, there are striking similarities regarding the nature of the sensory information that is conveyed to the spinal cord and VNC and how it is processed and used to control movement.

## SENSORY FEEDBACK, CENTRAL PATTERN GENERATORS, AND DESCENDING MOTOR CONTROL

**Figure 1:**
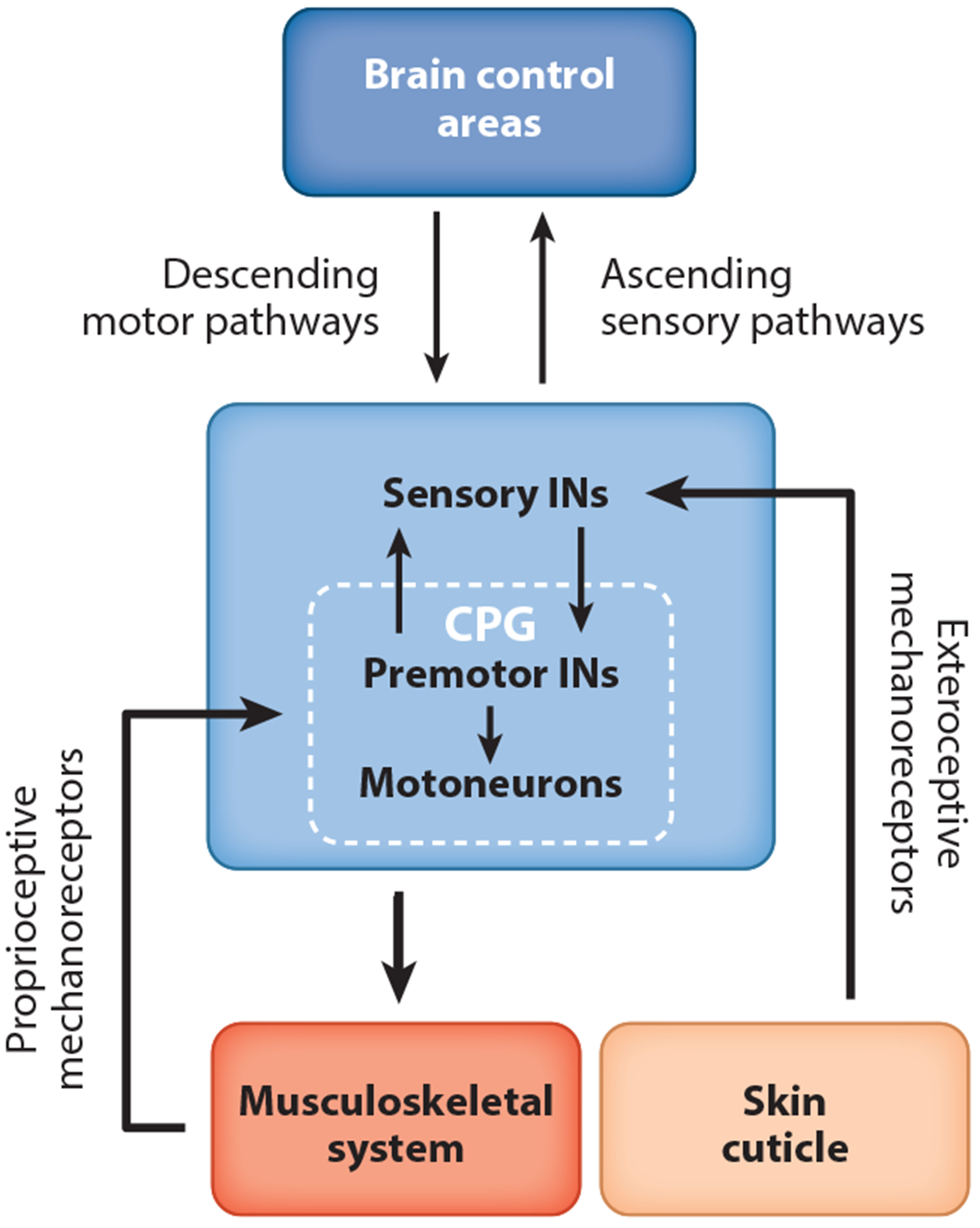
General organization of somatosensory feedback pathways in insects and mammals. Schematic shows the overall organization of sensory feedback pathways and local sensorimotor networks that they control in the spinal cord of vertebrates and ventral nerve cord of invertebrates. Note the overall conserved structure of the pathways mediating sensory feedback and motor control present in arthropods and chordates. Abbreviations: CPG, central pattern generator; IN, interneuron. Illustration provided by Yolanda Leenders-Goulding.

It was Charles Sherrington (1906) who first demonstrated a key role for sensory feedback in generating simple reflexes and locomotion. This led him to propose that motor behaviors are in their elemental form chains of sensorimotor reflexes. Subsequently, it was shown that simple rhythmic motor behaviors can be generated in the absence of sensory feedback by intrinsic neuronal networks (Brown 1911, Delcomyn 1980, Kiehn 2016) termed central pattern generators (CPGs). We now know that these CPG networks are malleable and can be reconfigured by sensory feedback to generate a wide range of rhythmic and nonrhythmic motor outputs (Grillner 2006). This has led to the view that naturalistic motor behaviors arise from the extensive interplay between local sensory feedback pathways and their associated CPG networks, which are subject to executive control by descending motor pathways from the brain (Figure 1).

**Figure 2:**
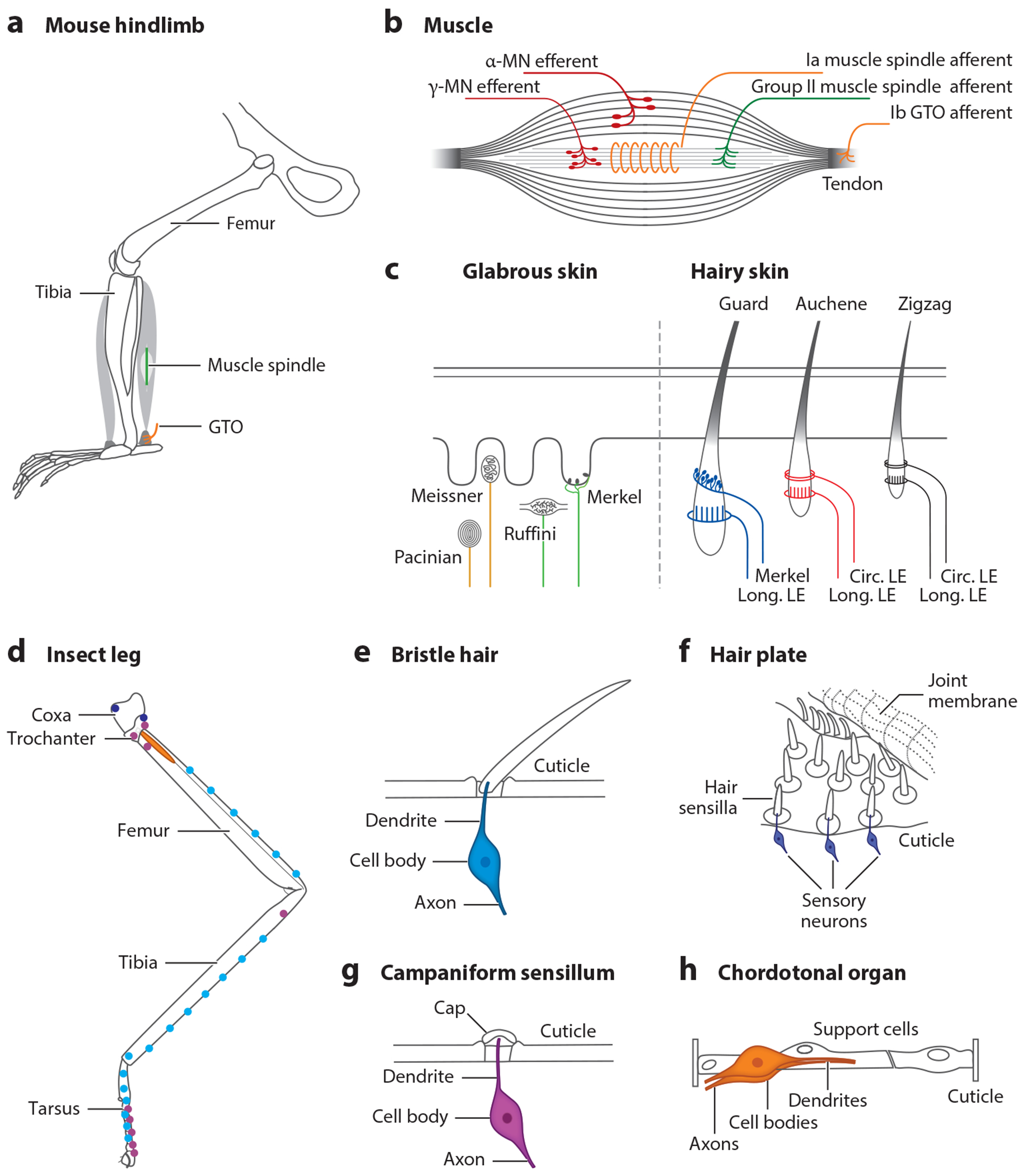
Anatomy of mechanosensory organs present in mammals and insects. (*a*) Schematic of a mouse hindlimb showing the location of the muscle spindle (*green*) that is associated with the intrafusal muscle bundle (*light gray*) of the gastrocnemius muscle and the Golgi tendon organ (*orange*) that innervates the ankle Achilles tendon. (*b*) Anatomy of striated muscles showing innervation of the intrafusal muscle fibers by γ-MNs and Ia and group II muscle spindle afferents that innervate the equatorial and polar regions of the intrafusal muscle fibers, respectively. Ib GTO afferents with characteristic flower spray endings innervate tendons (for details, see Matthews 2015). (*c*) Anatomy of cutaneous mechanoreceptors. The glabrous skin is innervated by rapidly adapting LTMR afferents, which are associated with Pacinian and Meissner mechanoreceptors, and slowly adapting LTMR afferents, innervating Merkel and Ruffini mechanoreceptors. The three hair types found in hairy skin are innervated by Merkel cell receptors and circular and longitudinal LEs (for details, see Abraira & Ginty 2013). (*d*) Schematic showing a standard insect leg exemplified by a phasmid middle leg. Colored elements and circles depict example locations of the exteroceptors and proprioceptors shown in panels *e–h*. Please note that the sensory neurons are depicted in the color that matches their localization on the schematized leg. (*e*) Bristle hair: The sensory neuron is activated by displacement of the bristle. (*f*) Hair plate: Sensory neurons innervating each hair are activated by mechanically bending the joint or releasing the hairs. (*g*) Campaniform sensillum: The sensory neuron is activated by deformations of the cuticle. (*h*) Chordotonal organ: Sensory neurons can be activated by position, velocity, acceleration, or vibration. Abbreviations: GTO, Golgi tendon organ; LE, lanceolate ending; LTMR, low-threshold mechanoreceptor; MN, motoneuron. Illustrations provided by Yolanda Leenders-Goulding and Sherylane Seeliger.

The primary sources of sensory feedback that animals use for motor control are (*a*) proprioceptors associated with the musculoskeletal system and (*b*) cutaneous low-threshold mechanoreceptors (LTMRs) that innervate the skin of vertebrates and mechanosensory exteroceptors in the cuticular tissue of insects (Figure 2). Proprioceptive mechanoreceptors provide kinesthetic information to the nervous system that registers body position and dynamics, while cutaneous mechanoreceptors or exteroceptors primarily monitor contact forces with the external environment. These two streams of somatosensory information generate a composite representation of the body and its interactions with its surroundings that is used for movement and for postural control.

### The Proprioceptive Machinery

All animals rely on proprioceptive feedback for accurate movement and postural control. This information is provided by specialized mechanoreceptors that function to monitor the forces acting on the musculoskeletal system and are tailored to the different body plans found in the animal kingdom.

#### Vertebrates.

Much of our current understanding of proprioception comes from studying the specialized proprioceptive organs present in the limbs of vertebrates and their associated sensory afferents (Matthews 2015). These organs are the (*a*) muscle spindles found in striated muscle that encode dynamic and static changes to muscle length and (*b*) Golgi tendon organs (GTOs) in tendons that measure the force being exerted by the associated muscle (Figure 2a). Muscle spindle afferents measure changes in the length of the non-force-producing intrafusal fibers in the muscle bed of striated muscles. These intrafusal fibers are innervated by gamma motoneurons that regulate their tension and modulate muscle spindle sensitivity during movement. This modulation of muscle spindle activity has the capacity to generate a sensory signal that is phase advanced, thus overcoming the limitations in feedback control that are caused by conduction delays, particularly those that occur in the long sensory axons of limb muscles. By contrast, the firing rates of Ib GTO afferents provide a direct measure of muscle force as GTOs are not innervated (Houk & Henneman 1967, Prochazka & Gorassini 1998). In addition to these classical proprioceptors, there is evidence that slowly adapting and rapidly adapting joint afferents that report joint angle and LTMR afferents that sense skin stretch provide additional proprioceptive information to the motor system in mammals (Proske & Gandevia 2012). Muscle spindle and GTO proprioceptors that are functionally adapted to the appendicular musculature of terrestrial vertebrates are largely lacking in swimming vertebrates. They instead possess specialized intrinsic spinal proprioceptors that register axial bending, including edge cells in lampreys (Grillner et al. 1984) and intraspinal lateral proprioceptors in jawed fish (Picton et al. 2021). Polymodal cerebrospinal fluid–contacting neurons that line the central canal of the spinal cord also serve as a source of proprioceptive feedback to the swimming CPG by reporting axial curvature (Wyart et al. 2023).

#### Invertebrates.

Insects also possess specialized proprioceptors, most prominently hair plates (HPs), located on the cuticle at joints between segments, and chordotonal organs (COs), located internally in one segment and connecting to a neighboring segment via a ligament (Figure 2). COs encode position, velocity, and acceleration and are therefore functionally analogous to Ia muscle spindles (Hofmann et al. 1985). They do so by measuring the physical displacement of segments relative to each other. Campaniform sensilla (CS), a third proprioceptor type, sense strain on the cuticle arising either from external forces or from resisted muscle forces and thus appear to be the functional correlate of GTOs in mammals (Dickinson et al. 1997, Tuthill & Azim 2018). While GTOs have been described to exclusively respond to force increases, CS have recently been reported to also encode force decreases (Harris et al. 2022). Despite these morphological and mechanical differences, a high degree of functional overlap exists in the proprioceptive information provided to the vertebrate and invertebrate nervous systems and how it is used to control specific aspects of movement.

### Proprioception and Movement

Much of what we know about the role of proprioception in movement comes from studies in cats and rodents, and to a lesser degree in primates and humans. Loss-of-function mutations involving Piezo2, the primary mechanosensitive channel present in proprioceptors, underline the importance of proprioception for coordinated movement (Chesler et al. 2016, Takeoka et al. 2014, Woo et al. 2015). Proprioceptive feedback modulates the force of movement, and it stabilizes the limb in response to external and postural perturbations (Donelan et al. 2009, Hasan & Stuart 1988, Windhorst 2007) and self-generated motor activity (Fink et al. 2014). Muscle afferent feedback contributes to the initiation of locomotion by signaling the position of the hip to initiate the swing phase (Grillner & Rossignol 1978). GTO feedback stabilizes the limb during walking by inhibiting flexor activity during stance (Conway et al. 1987, Hiebert et al. 1996, Whelan et al. 1995) but then initiates the transition from stance to swing when the load-related signal from ankle Ib afferents at the end of stance is reduced during walking (Pearson 2008). Proprioceptors also play a prominent role in coordinating limb synergies by organizing the pattern of motor activity at more distal joints. Mice lacking muscle spindles exhibit marked changes in the timing of ankle flexor muscle activity during walking (Akay et al. 2014). This is further exacerbated during swimming due to the additional loss of GTO feedback, resulting in synchronous flexor activation along the length of the leg. While less is known about feedback from group II muscle spindles, by providing information about slow movements and limb position (Banks et al. 2021), they are thought to play an important role in weight-bearing and postural control. The recent molecular profiling of proprioceptor subtypes (Oliver et al. 2021, Wu et al. 2021) opens up the possibility of using genetic manipulations to assess the contribution that specific proprioceptor types make to motor control.

Studies in invertebrate models have revealed similar roles for proprioception in insect locomotion, for both walking and flight (Büschges & Ache 2025, Frye & Dickinson 2004). Proprioceptive feedback modulates the magnitude of motor activity, that is, force, and it contributes to postural control by stabilizing the limb segments in response to gravity as well as external and postural perturbations. It also regulates the timing and duration of stance and swing. In particular, sensory feedback from HP and CO position– and movement-sensitive afferents plays a prominent role in controlling and initiating stance and swing (Bässler 1977). Information about the position and movement of the coxa and the tibia that comes from HP afferent and femoral CO feedback, respectively, is used to control stance activity and the transition from stance to swing (Bässler & Büschges 1998). Moreover, when afferent feedback from these two sense organs is modified so that it continuously reports one fixed joint position, the pattern of motor activity stays locked in one phase of the step cycle. Further evidence that proprioceptive feedback is important for controlling leg stepping also comes from the observation that optogenetically silencing the femoral CO sensory neurons affects all phases of the leg stepping cycle, that is, swing, stance, and the transitions between these two phases (Chockley et al. 2022). The selective stimulation of both sensory systems in otherwise deafferented semi-intact or reduced preparations during walking reveals that these proprioceptors have access to the leg locomotor CPGs and determine their phase of activity (Büschges & Ache 2025, Mantziaris et al. 2020). Similar to what has been described in cats and mice, movement- and load-related feedback regulates intra- and interjoint coordination in stick insects to facilitate motor synergies, including those involving the coxa-trochanter and femur-tibia joints (Hess & Büschges 1999).

Insect flight is also heavily dependent on proprioceptive feedback (Agrawal & Tuthill 2022, Mantziaris et al. 2020). Orthopteran insects (hawkmoths, locusts, crickets, and dragonflies) use low wing beat frequencies for flight, in which wing upstroke and downstroke movements are driven independently of each other by muscle activation. Of the four sensory systems that control each wingbeat cycle in locusts, two have been analyzed at a circuit level in the VNC. Thoracic wing stretch receptors that are located at the base of each wing (Gettrup 1965) act to signal the upper position of the wing (Pabst 1965). Their activity raises the wing beat frequency via a tonic excitatory influence on wing depressor motoneurons (Pearson & Ramirez 1990), which is mediated by direct connections between the stretch receptors and wing motoneurons as well as indirect connections involving the flight CPG (Mantziaris et al. 2020). Knob-shaped sensory organs on the anterior base of each wing, called wing tegulae, signal the downstroke of the wing (Wolf & Pearson 1988). The hindwing tegula also initiates wing elevator activity to increase wing beat frequency, again via direct and disynaptic connections to motoneurons and inputs to the CPG (Mantziaris et al. 2020). The underlying circuitry for the two other proprioceptor types that affect flight motor pattern has not been mapped. These are the wing CS located on the wing veins (Gettrup 1965) that stabilize flight by signaling cuticular stress arising from wing deformation, and proprioceptors located at the base of the wing muscles that are activated by flight muscle contractions and provide feedback to the flight circuitry (Stevenson 1996). In flies, bees, and wasps, where wingbeat frequencies are too high for individual stroke control, the oscillatory properties of the wing-bearing thorax and its musculoskeletal system are driven by the calcium-dependent asynchronous activation of wing muscles (Gordon & Dickinson 2006). This form of flight also depends on sensory feedback to control wing beat and task-dependent activity changes (Heide 1979). Dipteran insects (flies and blow flies) also use cycle-to-cycle CS feedback from the haltere to regulate motoneuron activity for both the halteres and wings (Dickerson et al. 2019, Verbe et al. 2024).

### Local Circuits for Proprioception

**Figure 3:**
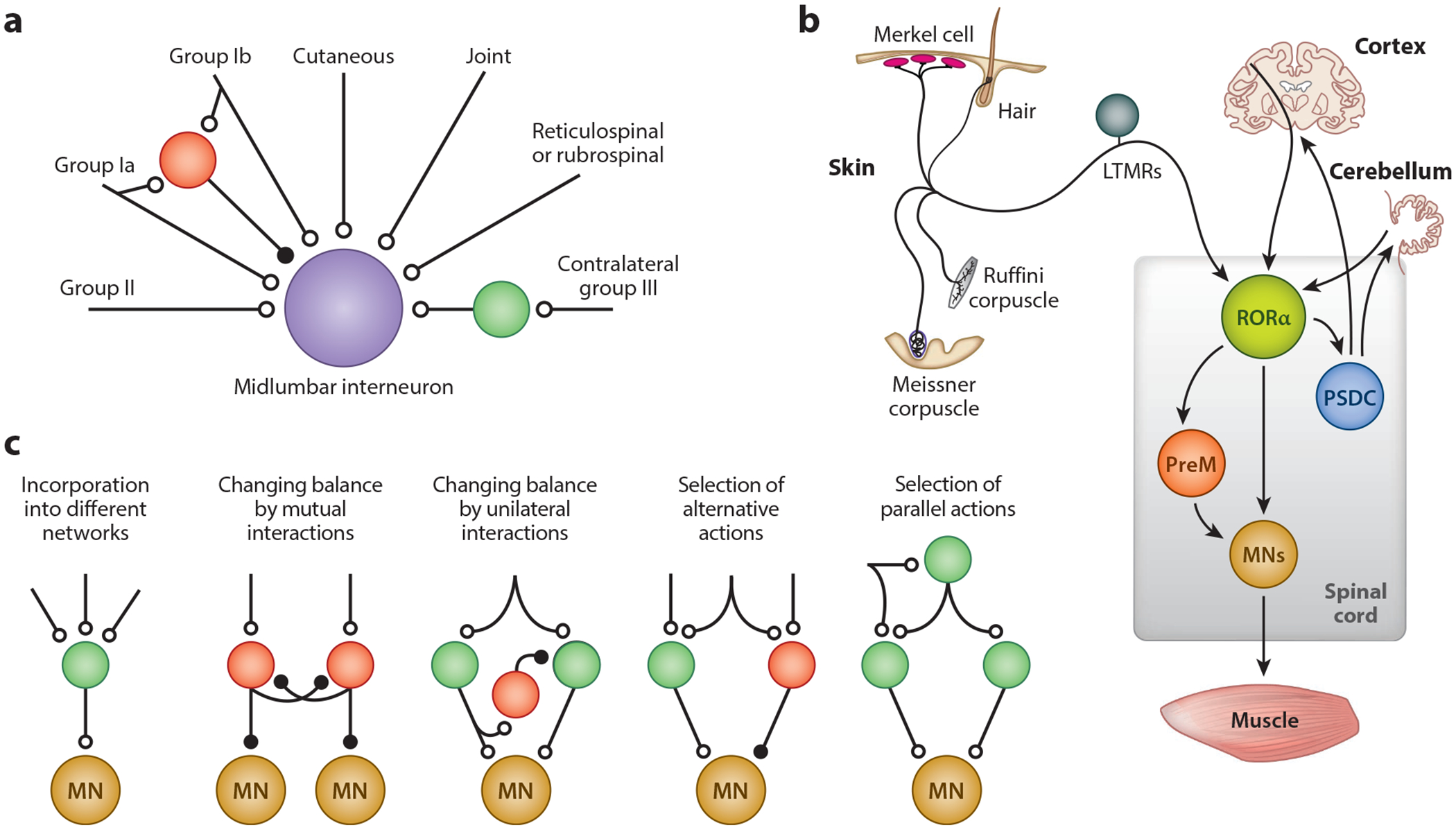
Organizational features of the spinal sensorimotor networks that control movement. (*a*) Schematic showing convergent inputs to group II midlumbar interneurons. This population can be subdivided into neurons that receive monosynaptic inputs from ventral descending pathways (reticulospinal and vestibulospinal) or from dorsal descending pathways (rubrospinal and corticospinal). Panel *a* adapted with permission from Edgley (2001) (for additional details, see Edgley 2001). (*b*) Schematic of RORα interneuron connectivity showing that these neurons transmit and integrate sensory feedback from glabrous skin LTMRs, and they receive convergent excitation from descending motor pathways. This connectivity underpins their role in generating corrective limb movements. Panel *b* adapted from Bourane et al. (2015b) (for additional details, see Bourane et al. 2015b). (*c*) Schematic showing task- and state-dependent reconfigurations of PreM interneuron networks that occur in the mammalian spinal cord. These reconfigurations generate the different task-dependent patterns of motor activity that underlie all spinally driven motor behaviors. Panel *c* adapted with permission from Jankowska (2001) (for additional details, see Jankowska 2001). Open circles represent excitatory synapses, and closed circles indicate inhibitory synapses in panels *a* and *c*. Abbreviations: LTMR, low-threshold mechanoreceptor; MN, motoneuron; PreM, premotor interneuron; PSDC, postsynaptic dorsal column; RORα, retinoic acid orphan receptor alpha. Illustrations by Yolanda Leenders-Goulding.

Much of what we know about proprioceptive pathways in the spinal cord comes from studies in the cat. Proprioceptive information is distributed widely throughout the spinal cord, where it regulates motor activity either directly via monosynaptic Ia inputs to motoneurons or indirectly via the recruitment of Ia, Ib, and group II interneurons. Direct excitatory Ia muscle spindle input to homonymous motor neurons is important for postural control, with motoneurons innervating antigravity (extensor) muscles receiving the strongest monosynaptic Ia input (Taylor & Gottlieb 1985). Ia afferent excitation of motoneurons is also spread over synergistic motoneuron pools (Eccles et al. 1957a), which is functionally well-suited to binding muscle synergies. All other proprioceptive input to motoneurons is indirect via different classes of neurons that are interposed in the proprioceptive feedback pathways to motoneurons (Jankowska 1992). In the cat, these neurons have been classified as Ia, Ib, and group II interneurons according to their predominant input. However, they do receive a complex matrix of weighted connections, suggesting they can be recruited to generate a wide range of motor outputs (Figure 3). Take, for example, reciprocal Ia inhibitory neurons that are strongly activated by Ia afferents (Eccles et al. 1957a,b; Feldman & Orlovsky 1975; Hultborn et al. 1971) and secure reciprocal inhibition between antagonist flexor and extensor muscle groups (Britz et al. 2015, Kiehn 2016, Zhang et al. 2014). They receive inputs from multiple pathways, including corticospinal, rubrospinal, reticulospinal, vestibulospinal, and Ia pathways (Jankowska 1992), thereby integrating a range of sensory and descending information to coordinate the flexion-extension behaviors that underlie limb-driven motor behaviors. Ib inhibitory neurons (IbINs) that are innervated by GTO afferents have also been extensively studied (Jankowska 1992). IbINs provide feedback to the autogenic motoneurons that innervate the same muscles from which their GTO afferents arise. Interestingly, autogenic GTO activity alone is insufficient to recruit IbINs, indicating that IbIN activity requires convergent input from additional sensory and descending pathways (Jankowska 1992). As such, IbINs are well-placed to facilitate functional muscle synergies during movement. IbINs also contribute to state-dependent reflex changes during ongoing movement, particularly those seen in extensor and bifunctional muscles during stance (McCrea 2001, Pearson 2008). Moreover, they receive Ia feedback that likely acts together with Ib input to modulate IbIN excitability in response to changes in muscle length (Jankowska & McCrea 1983). Some interneurons with group II input also receive convergent input from Ia and Ib afferents, the effects of which can be coexcitatory, coinhibitory, or mixed (Edgley 2001, Jankowska 1992) (see Figure 3). These examples highlight how sensory feedback from different proprioceptive pathways is integrated at the premotor interneuronal level.

The locomotor systems generating flight and walking signals from proprioceptive sense organs that are associated with the legs and wings are processed in spatially defined areas of the VNC (Lesser et al. 2024, Ramirez & Pearson 1988). The shortest connections between sensory neurons and motoneurons are the direct excitatory pathways (Burrows 1987, Pearson et al. 1976). Parallel to these are di- and polysynaptic pathways comprising connections to local CPG interneurons and intercalated non-CPG interneurons (Büschges & El Manira 1998). The most detailed circuit picture of connectivity between individual proprioceptive sensory neurons, identified local interneurons, and motoneurons comes from the locust metathoracic ganglion. These circuits comprise four neuronal populations: the midline group, the anteriolateral group, the anteriomedial group, and local nonspiking interneurons (Burrows 1996). The roles that individual neurons in the metathoracic ganglion play in processing sensory information from HP, CO, and CS afferents to motoneurons have largely been defined (Burrows 1996). Further insights into the functional organization of these sensorimotor circuits in the insect VNC are now emerging from the detailed neural circuit analysis of the fruit fly connectome (Agrawal et al. 2020, Chen et al. 2021, Lesser et al. 2024).

Studies in the stick insect have shed light on the insect leg muscle control system in the VNC (Gebehart & Büschges 2024). The motor activity this network generates for ongoing locomotion (Kittmann et al. 1996, Wolf & Büschges 1995) and postural control (Büschges & Wolf 1995, Gebehart & Büschges 2024, Gebehart et al. 2021, Sauer et al. 1996) arises from the relative weightings of synaptic drive within the network. Conjoint action within these parallel pathways serves gain control and the reversal of postural reflexes at rest that assist reflexes during locomotion (Bässler 1976, Driesang & Büschges 1996), a phenomenon that is also seen in mammals, including humans (Conway et al. 1987, Duysens et al. 1990, Forssberg et al. 1975, Pearson & Collins 1993), and other arthropods (DiCaprio & Clarac 1981). As noted with the spinal cord, interneuronal feedback pathways in the VNC appear to be multimodal with respect to the proprioceptive signals they process. For example, sensorimotor pathways involved in the control of the tibia, that is, tibial flexor and extensor motoneurons, co-process information about movement and force/load (Gebehart et al. 2021). Lateral interactions within and between different sensory modalities underlie gain control by sensorimotor influences. Interneuron-to-interneuron interactions between premotor interneurons are thought to facilitate this convergence in sensorimotor processing (Gebehart et al. 2021), a principle of sensory information processing that has also been documented in the mammalian spinal cord (Jankowska 2022).

## EXTEROCEPTIVE SENSORY FEEDBACK

External forces that act on the body are also used for the dynamic control of movement. These contact forces are monitored by specialized exteroceptive/cutaneous mechanoreceptors (Figure 2) and are used to generate a range of corrective and protective reflex behaviors.

### Cutaneous Mechanoreception in Vertebrates

Much of what we know about the exteroceptive somatosensory system in vertebrates comes from the characterization of LTMRs in the hairy and glabrous skin of mammals and their associated sensory afferents (Abraira & Ginty 2013, Brown 1981) (Figure 2c). These specialized LTMRs respond to different light touch features and relay this information to the spinal cord and brain where it is used for motor control and tactile perceptive tasks. LTMR afferents have axons that branch and terminate in the LTMR-recipient zone of the dorsal horn. Their axon terminals have a characteristic morphology, and they are organized topographically in different dorsal laminae to form a 3D map of the body surface (Abraira & Ginty 2013, Brown 1981, Schouenborg 2002). For example, Meissner afferents primarily terminate in lamina IIi/III, whereas Pacinian terminals are located slightly deeper in lamina III/IV (Brown 1981). Hair follicle afferents also terminate in a laminar-specific manner in columns that are tiled to produce a somatotopic map of the body surface (Li et al. 2011). There is, however, a significant amount of overlap in the termination zones of different LTMR afferent types, with sensory interneurons in the LTMR-recipient zone typically receiving input from more than one LTMR subtype (Abraira et al. 2017, Bourane et al. 2015b).

The 3D organization of sensory inputs from different LTMR subtypes in the dorsal horn has important implications for the central processing of mechanosensory information. First, it reveals that different LTMR modalities partially overlap at the level of the first central synapse, which is important for the functional organization of sensorimotor reflexes (Gatto et al. 2021). Second, cutaneous terminations in the superficial dorsal horn, which includes the LTMR-recipient zone, form a somatotopic map that closely aligns with the musculotopic map present in the deep dorsal horn, where reflex encoder (RE) neurons in lamina IV/V form sensorimotor modules that cojoin areas of the skin with the motoneurons and muscles that withdraw the same area of the skin away from the stimulus (Schouenborg 2002). These sensorimotor modules represent the functional building blocks that the spinal cord, cerebellum, and cortex use to generate the map of the body and use it to coordinate movement.

### The Spinal Circuitry for Cutaneous Transmission

Cutaneous sensory pathways access the spinal premotor CPG network via a variety of disynaptic and polysynaptic pathways (Edgley & Wallace 1989, Engberg 1964, Hagbarth 1952, Jankowska 1992). To date, the spinal circuits that provide cutaneous sensory information to the motor system remain largely unmapped. This is rapidly changing as a result of studies aimed at molecularly identifying and characterizing sensory interneuron cell types in the dorsal horn (Abraira et al. 2017, Bröhl et al. 2008, Del Barrio et al. 2013, Gross et al. 2002, Häring et al. 2018, Müller et al. 2002, Sathyamurthy et al. 2018). For example, RORα sensory interneurons that are innervated by LTMR afferents form synaptic contacts onto excitatory V2a and V0c CPG neurons in the ventral horn (Figure 3b), while other functional studies reveal specific roles for particular sensory interneuron populations in transmitting and gating different streams of cutaneous mechanosensory information (Gatto et al. 2021, Koch et al. 2018).

Many of the sensory interneurons that transmit cutaneous information are innervated by descending projection neurons, one example being the innervation of RORα-expressing excitatory interneurons by corticospinal and vestibulospinal projection neurons (Bourane et al. 2015a) (Figure 3b). Interneurons in lamina V that are monosynaptically connected to motoneurons have also been identified in the mouse (Levine et al. 2014). These neurons receive sensory and corticospinal input and may therefore be the functional equivalents of RE neurons that have been described in the rat (Schouenborg 2002) or lamina IV/V interneurons in the cat that receive group II and cutaneous inputs (Jankowska 1992). Interestingly, the innervation of sensory interneurons in the LTMR-recipient zone by descending brain projections mirrors the connectivity previously described for identified cat premotor interneurons that are located more ventrally in the spinal cord (Edgley & Jankowska 1987, Jankowska 1992), and it argues that descending control of movement is mediated in large part by descending motor control pathways coopting the sensorimotor circuitry of the spinal cord to generate a specific motor action and/or suppress alternative motor behaviors, for example, protective spinal reflexes.

### The Role of Cutaneous Afferents in Mammalian Locomotion

Sensory feedback from cutaneous mechanoreceptors, in addition to activating rhythmic behaviors, for example, scratching, grooming, and paw shake (Rossignol et al. 2006), plays a prominent role in the adaptive control of movement and the execution of skilled motor tasks such as precision grasping (Witney et al. 2004). Cutaneous feedback is required for corrective movements, proper foot placement, and demanding locomotor tasks such as incline walking and grasping (Rossignol et al. 2006). It also helps stabilize the limb during stance on uneven surfaces (Zehr et al. 1998). Cutaneous feedback contributes to flight control in bats and birds by sensing airflow across the wings (Brown & Fedde 1993, Sterbing-D’Angelo et al. 2011). In bats this information is provided by mechanoreceptors that are associated with sensory hairs on the bat wing, removal of which decreases flight maneuverability (Marshall et al. 2015, Sterbing-D’Angelo et al. 2011).

Cutaneous feedback is particularly important for corrective movements during ongoing locomotion such as the vestibulospinal corrective reflexes that enable walking on an uneven surface or narrow beam (Bourane et al. 2015b) and the stumbling corrective reflex (Forssberg 1979, Forssberg et al. 1975). A key feature of this feedback is its modulation in a state- and task-dependent manner, best exemplified by the stumbling corrective reflex where touching the paw dorsum during swing serves to flex the limb and prevent stumbling, while the same stimulus during stance leads to extension (Forssberg 1979). Further evidence that cutaneous sensory pathways contribute to corrective motor behaviors comes from genetic studies in the mouse. Ablation of excitatory interneurons in the LTMR-recipient zone that are innervated by innocuous mechanoreceptors results in a marked increase in falls and slips on the narrow beam (Bourane et al. 2015b, Gatto et al. 2021). Interestingly, studies in humans have shown that LTMRs in the glabrous skin of the feet contribute to the corrective movements that are necessary for maintaining balance (Perry et al. 2000). Moreover, reduced feedback from the soles of patients with peripheral neuropathies and Parkinson’s disease is thought to contribute to an increase in slips and falls (Prätorius et al. 2003).

### Exteroceptive Sensory Feedback in Insects and Its Role in Locomotion

Exteroception in insects is mediated by cuticular mechanoreceptors that sense touch (Burrows 1996) and that are particularly abundant on the thorax, wings, and legs. Among the receptors that report touch are tactile hairs, CS, and the so-called canal receptors on the pad of the tarsus (Kendall 1970, Laurent & Hustert 1988). These mechanoreceptors provide sensory feedback about touch at rest or when there is contact with an object during active movements and locomotion. Exteroceptive sensory feedback in insects drives corrective motor behaviors and protective reflexes in a task-dependent fashion, in much the same way that cutaneous feedback has been shown to in mice and cats (Bourane et al. 2015b, Forssberg et al. 1975). For example, exteroceptive signals from the tarsus initiate elevation of the leg upon touch or the electrical activation of the respective afferents at rest, whereas during walking, the same stimulus has no effect during stance but instead induces an additional short leg protraction during swing (Wolf 1992). Likewise, stimulation of tactile hairs at any location along a leg’s segment generates coordinated avoidance reflexes that move the leg away from the stimulus site (Siegler & Burrows 1986). Studies in *Drosophila* have revealed that mechanosensory bristles on the leg are important for reflex behaviors such as back-leg kicking and avoidance movements (Li et al. 2016, Medeiros et al. 2024). Interestingly, the initial movement of the leg is controlled by the VNC and does not depend on descending motor commands from the fly brain (Medeiros et al. 2024), thereby demonstrating the role that short-latency interactions at the level of the VNC play in generating rapid corrective and protective reflexes. Exteroceptive sensory signaling is also important for flight control in insects, where it adapts the flight-related motor activity needed for coordinated and stable flight (Dickinson 1999, Elson 1987, Wendler 1978).

## INHIBITION AND THE GATING OF SENSORY FEEDBACK

Sensory afferent pathways are organized so that incoming sensory information is dynamically regulated in a state- and task-dependent manner. The regulation of sensory information is mediated in large part by the postsynaptic inhibitory interneurons that pattern output of the locomotor CPG (see discussion above, and for details, see Jankowska 1992) and presynaptic inhibitory neurons that gate the transmission of sensory information in the VNC and spinal cord (Clarac & Cattaert 1996, Rudomin & Schmidt 1999). Presynaptic inhibition is largely mediated by GABAergic neurons that form axon-axonic synapses on the presynaptic terminals of sensory afferents (Burrows & Laurent 1993, Eccles et al. 1963, Gray 1962, Sauer et al. 1997, Watson 1992), termed GABApre neurons, that are thought to attenuate sensory transmission by a mechanism called primary afferent depolarization (PAD) by which neurotransmitter release from the sensory axon terminal is reduced (Rudomin & Schmidt 1999). In some instances, these GABApre neurons also form postsynaptic inhibitory synapses on the dendrites of their postsynaptic targets, demonstrating that presynaptic and postsynaptic inhibitory pathways are able to work together to regulate sensory transmission (Maxwell & Riddell 1999, Solodkin et al. 1984).

The control of sensory feedback by PAD endows motor systems with a high degree of behavioral flexibility. Presynaptic inhibition cancels out self-generated sensory information that is generated during vertebrate movement (Fink et al. 2014), and it suppresses reflexes that have the potential to disrupt any ongoing motor program (Seki et al. 2003). Presynaptic inhibition also plays a key role in action selection by ensuring specific motor behaviors are expressed while others are suppressed (Gaudry & Kristan 2009). Examples of presynaptic inhibition in action during movement include the reduction in the strength of synaptic inputs from muscle spindle afferents during walking, which attenuate the stretch reflex (Dueñas & Rudomin 1988), and the state-dependent reversal of GTO reflexes in extensor muscles during walking versus rest (Conway et al. 1987, Pearson & Collins 1993). Genetic studies in mice show that the degradation of presynaptic inhibition onto proprioceptive afferents disrupts ongoing locomotion (Koch et al. 2017) and skilled reaching movements (Fink et al. 2014), while the loss of GABA_A_ inhibition directed toward cutaneous LTMR afferents alters tactile sensitivity in the glabrous skin (Zimmerman et al. 2019). While PAD activity in sensory afferents is thought to be primarily inhibitory in nature, a recent study has shown that it can also facilitate sensory transmission by promoting spike propagation through the branch points of sensory axons in the spinal cord where branch point failure can occur (Hari et al. 2022).

Work in the cat spinal cord has provided key insights into many of the salient features of presynaptic inhibition (reviewed in Rossignol et al. 2006, Rudomin & Schmidt 1999). Most notably, PAD is engaged by multiple sources, including the locomotor CPG, other sensory afferents, and descending spinal pathways. This organizational structure ensures the induction of PAD is coordinated in a task- and phase-dependent manner. One key feature of PAD is the different patterns of recruitment that occur in the different sensory fiber types (Rudomin & Schmidt 1999). For example, whereas flexor Ia afferents induce PAD in extensor and flexor muscle spindles and flexor GTO fibers, extensor Ia afferents do not. When the synergistic or antagonistic actions of cutaneous afferents and descending supraspinal pathways are included, the picture that emerges is one of a complex matrix of task-dependent PAD recruitment by descending and sensory pathways and by the elements of the CPG. Moreover, presynaptic inhibitory circuits display a strict somatotopic organization that is also modality specific, one example being the segmental induction of PAD in cutaneous and Ib afferents by spatially distinct populations of neurons in the somatosensory cortex (Andersen et al. 1962). Recent studies show that distinct spinal inhibitory interneuron populations differentially inhibit light touch, pain, and proprioceptive transmission (Bourane et al. 2015a, Kardon et al. 2014, Koch et al. 2017). Some of these populations express neuropeptides such as neuropeptide Y and dynorphin, whose actions inhibit the flow of sensory information in the cord, thereby adding another layer of inhibitory control to the spinal circuits that process sensory information (Acton et al. 2019, Kardon et al. 2014). Finally, there is growing evidence that identified premotor CPG interneurons are able to gate sensory transmission by presynaptic inhibitory mechanisms, either directly or indirectly in the case of the V3 INs (Lin et al. 2023), and directly in the case of a subpopulation of V1 INs that form presynaptic synapses onto cutaneous afferents (G. Gatto & M. Goulding, unpublished data).

Presynaptic inhibition has also been extensively studied in invertebrates, including insects, where it modulates proprioceptive feedback both in the context of motor control (Clarac & Cattaert 1996) and in the processing of courtship-related sensory information (Poulet & Hedwig 2002). Similar to what is seen in the mammalian spinal cord, sensory feedback in invertebrates is controlled by PAD during ongoing locomotor activity (Büschges & Wolf 1999, Wolf & Burrows 1995), and it can be induced in a cross-modal manner by other sensory afferent types. For example, the leg position–sensitive femoral CO in the stick insect exhibits PAD from CS and coxal HP proprioceptors, or from CS and femoral CO proprioceptors and vice versa (Gebehart et al. 2021, Stein & Schmitz 1999). While the action of presynaptic inhibition has been studied in some detail, the identity of the neurons involved is only known in one case, these being the target sensory neurons (Poulet & Hedwig 2002). These findings argue that the task- and context-dependent gating of sensory information by presynaptic inhibition that is driven by local circuits and descending pathways is a shared feature of the vertebrate and invertebrate sensorimotor systems. This conservation of presynaptic inhibitory mechanisms across the animal kingdom points to an early evolutionary origin of presynaptic inhibition. Relatedly, it has been suggested that the ancestral function of the corticospinal tract was to control presynaptic inhibition in the spinal cord, with its role in fine motor control only emerging later.

## CONCLUSIONS AND FUTURE DIRECTIONS

Many of the general principles governing the sensory control of movement are now known and are described in this review. Much less is known about the organization of the sensorimotor networks in the spinal cord and VNC that process this sensory information and how they are reconfigured by sensory feedback and descending motor commands. What we do know is that these control pathways act on local interneuron networks in the spinal cord and VNC, reshaping them in a task-dependent manner whereby certain connections become dominant and others less so; however, defining the details of these dynamic changes remains a challenge for the field.

Recent progress has been made on the molecular profiling and characterization of interneuron and sensory cell types in the mouse spinal cord and *Drosophila* VNC, thus opening the door to mapping and functionally interrogating these networks at a cellular level. In *Drosophila*, these efforts are being augmented with electron microscopic anatomical reconstruction studies to generate a cell type connectivity map of the VNC, something that Dallmann et al. (2024) have now taken advantage of to characterize a population of GABAergic interneurons in the VNC that provide presynaptic inhibition to a subpopulation of proprioceptive sensory neurons during walking and grooming. What is still lacking is a dynamic view of these networks in action during movement so that one can better understand how they are reconfigured at a cellular level. This will require the large-scale monitoring of neural activity during different motor tasks, something that is potentially possible with the newly available genetic sensors and recording technologies that allow one to monitor the activity of large numbers of neurons during ongoing behavior.

Among the many additional questions that still need answering are the relative contributions that specific sensory modalities make to somatosensation and motor control. Another issue is how proprioceptive information is encoded and used to create the body schema the nervous system then uses to control movement. Other outstanding issues include the need to define where and how exteroceptive and proprioceptive sensory feedback pathways intersect with the different systems for descending motor control. Related to this is the question of how proprioceptive and exteroceptive information is transmitted and represented at different levels in the nervous system. These and many other questions are essential for understanding how complex nervous systems that are built around movement are organized and operate.

## References

[R1] Abraira VE, Ginty DD. 2013. The sensory neurons of touch. Neuron 79(4):618–39 23972592 10.1016/j.neuron.2013.07.051 PMC3811145

[R2] Abraira VE, Kuehn ED, Chirila AM, Springel MW, Toliver AA, 2017. The cellular and synaptic architecture of the mechanosensory dorsal horn. Cell 168(1–2):295–310.e19 28041852 10.1016/j.cell.2016.12.010 PMC5236062

[R3] Acton D, Ren X, Di Costanzo S, Dalet A, Bourane S, 2019. Spinal neuropeptide Y1 receptor-expressing neurons form an essential excitatory pathway for mechanical itch. Cell Rep. 28(3):625–39.e6 31315043 10.1016/j.celrep.2019.06.033 PMC6709688

[R4] Agrawal S, Dickinson ES, Sustar A, Gurung P, Shepherd D, 2020. Central processing of leg proprioception in *Drosophila*. eLife 9:e60299 33263281 10.7554/eLife.60299 PMC7752136

[R5] Agrawal S, Tuthill JC. 2022. The two-body problem: proprioception and motor control across the metamorphic divide. Curr. Opin. Neurobiol 74:102546 35512562 10.1016/j.conb.2022.102546 PMC9167723

[R6] Akay T, Tourtellotte WG, Arber S, Jessell TM. 2014. Degradation of mouse locomotor pattern in the absence of proprioceptive sensory feedback. PNAS 111(47):16877–82 25389309 10.1073/pnas.1419045111 PMC4250167

[R7] Andersen P, Eccles JC, Sears TA. 1962. Presynaptic inhibitory action of cerebral cortex on the spinal cord. Nature 194:740–41 13861184 10.1038/194740a0

[R8] Banks RW, Ellaway PH, Prochazka A, Proske U. 2021. Secondary endings of muscle spindles: structure, reflex action, role in motor control and proprioception. Exp. Physiol 106(12):2339–66 34676617 10.1113/EP089826

[R9] Bässler U. 1976. Reversal of a reflex to a single motoneuron in the stick insect *Çarausius morosus*. Biol. Cybernet 24(1):47–49

[R10] Bässler U 1977. Sense organs in the femur of the stick insect and their relevance to the control of position of the femur-tibia-joint. J. Comp. Physiol 121(1):99–113

[R11] Bässler U, Büschges A. 1998. Pattern generation for stick insect walking movements—multisensory control of a locomotor program. Brain Res. Rev 27(1):65–88 9639677 10.1016/s0165-0173(98)00006-x

[R12] Bourane S, Duan B, Koch SC, Dalet A, Britz O, 2015a. Gate control of mechanical itch by a subpopulation of spinal cord interneurons. Science 350(6260):550–54 26516282 10.1126/science.aac8653 PMC4700934

[R13] Bourane S, Grossmann KS, Britz O, Dalet A, Del Barrio MG, 2015b. Identification of a spinal circuit for light touch and fine motor control. Cell 160(3):503–15 25635458 10.1016/j.cell.2015.01.011 PMC4431637

[R14] Britz O, Zhang J, Grossmann KS, Dyck J, Kim JC, 2015. A genetically defined asymmetry underlies the inhibitory control of flexor-extensor locomotor movements. eLife 4:e04718 26465208 10.7554/eLife.04718 PMC4604447

[R15] Bröhl D, Strehle M, Wende H, Hori K, Bormuth I, 2008. A transcriptional network coordinately determines transmitter and peptidergic fate in the dorsal spinal cord. Dev. Biol 322(2):381–93 18721803 10.1016/j.ydbio.2008.08.002

[R16] Brown AG. 1981. The Organization of the Spinal Cord. Heidelberg, Ger.: Springer-Verlag

[R17] Brown RE, Fedde MR. 1993. Airflow sensors in the avian wing. J. Exp. Biol 179(1):13–30

[R18] Brown TG. 1911. The intrinsic factors in the act of progression in the mammal. Proc. R. Soc. B 84(572):308–19

[R19] Burrows M. 1987. Parallel processing of proprioceptive signals by spiking local interneurons and motor neurons in the locust. J. Neurosci 7(4):1064–80 3572474 10.1523/JNEUROSCI.07-04-01064.1987 PMC6568994

[R20] Burrows M. 1996. The Neurobiology of an Insect Brain. London: Oxford Univ. Press

[R21] Burrows M, Laurent G. 1993. Synaptic potentials in the central terminals of locust proprioceptive afferents generated by other afferents from the same sense organ. J. Neurosci 13(2):808–19 8426238 10.1523/JNEUROSCI.13-02-00808.1993 PMC6576656

[R22] Büschges A, Ache JM. 2025. Motor control on the move: from insights in insects to general mechanisms. Physiol. Rev 105(3):975–1031 39701070 10.1152/physrev.00009.2024

[R23] Büschges A, El Manira A. 1998. Sensory pathways and their modulation in the control of locomotion. Curr. Opin. Neurobiol 8(6):733–39 9914236 10.1016/s0959-4388(98)80115-3

[R24] Büschges A, Wolf H. 1995. Nonspiking local interneurons in insect leg motor control. I. Common layout and species-specific response properties of femur-tibia joint control pathways in stick insect and locust. J. Neurophysiol 73(5):1843–60 7623085 10.1152/jn.1995.73.5.1843

[R25] Büschges A, Wolf H. 1999. Phase-dependent presynaptic modulation of mechanosensory signals in the locust flight system. J. Neurophysiol 81(2):959–62 10036295 10.1152/jn.1999.81.2.959

[R26] Chen C, Agrawal S, Mark B, Mamiya A, Sustar A, 2021. Functional architecture of neural circuits for leg proprioception in *Drosophila*. Curr. Biol 31(23):5163–75.e7 34637749 10.1016/j.cub.2021.09.035 PMC8665017

[R27] Chesler AT, Szczot M, Bharucha-Goebel D, Čeko M, Donkervoort S, 2016. The role of PIEZO2 in human mechanosensation. N. Engl. J. Med 375(14):1355–64 27653382 10.1056/NEJMoa1602812 PMC5911918

[R28] Chockley AS, Dinges GF, Di Cristina G, Ratican S, Bockemühl T, Büschges A. 2022. Subsets of leg proprioceptors influence leg kinematics but not interleg coordination in *Drosophila melanogaster* walking. J. Exp. Biol 225(20):jeb244245 36268799 10.1242/jeb.244245

[R29] Clarac F, Cattaert D. 1996. Invertebrate presynaptic inhibition and motor control. Exp. Brain Res 112(2):163–80 8951385 10.1007/BF00227635

[R30] Conway BA, Hultborn H, Kiehn O. 1987. Proprioceptive input resets central locomotor rhythm in the spinal cat. Exp. Brain Res 68(3):643–56 3691733 10.1007/BF00249807

[R31] Dallmann CJ, Luo Y, Agrawal S, Chou GM, Cook A, 2024. Presynaptic inhibition selectively suppresses leg proprioception in behaving *Drosophila*. bioRxiv 2023.10.20.563322. 10.1101/2023.10.20.563322

[R32] Del Barrio MG, Bourane S, Grossmann K, Schüle R, Britsch S, 2013. A transcription factor code defines nine sensory interneuron subtypes in the mechanosensory area of the spinal cord. PLOS ONE 8(11):e77928 24223744 10.1371/journal.pone.0077928 PMC3817166

[R33] Delcomyn F 1980. Neural basis of rhythmic behavior in animals. Science 210(4469):492–98 7423199 10.1126/science.7423199

[R34] DiCaprio RA, Clarac F. 1981. Reversal of a walking leg reflex elicited by a muscle receptor. J. Exp. Biol 90(1):197–203

[R35] Dickerson BH, de Souza AM, Huda A, Dickinson MH. 2019. Flies regulate wing motion via active control of a dual-function gyroscope. Curr. Biol 29(20):3517–24.e3 31607538 10.1016/j.cub.2019.08.065 PMC7307274

[R36] Dickinson MH. 1999. Haltere-mediated equilibrium reflexes of the fruit fly, *Drosophila melanogaster*. Philos. Trans. R. Soc. B 354(1385):903–16 10.1098/rstb.1999.0442 PMC1692594 10382224

[R37] Dickinson MH, Hannaford S, Palka J. 1997. The evolution of insect wings and their sensory apparatus. Brain Behav. Evol 50(1):13–24 9209763 10.1159/000113318

[R38] Donelan JM, McVea DA, Pearson KG. 2009. Force regulation of ankle extensor muscle activity in freely walking cats. J. Neurophysiol 101(1):360–71 19019974 10.1152/jn.90918.2008

[R39] Driesang RB, Büschges A. 1996. Physiological changes in central neuronal pathways contributing to the generation of a reflex reversal. J. Comp. Physiol 179(1):45–57

[R40] Dueñas SH, Rudomin P. 1988. Excitability changes of ankle extensor group Ia and Ib fibers during fictive locomotion in the cat. Exp. Brain Res 70(1):15–25 3402561 10.1007/BF00271842

[R41] Duysens J, Trippel M, Horstmann GA, Dietz V. 1990. Gating and reversal of reflexes in ankle muscles during human walking. Exp. Brain Res 82(2):351–58 2286237 10.1007/BF00231254

[R42] Eccles JC, Eccles RM, Lundberg A. 1957a. The convergence of monosynaptic excitatory afferents on to many different species of alpha motoneurones. J. Physiol 137(1):22–50 10.1113/jphysiol.1957.sp005794 PMC1362996 13439582

[R43] Eccles JC, Eccles RM, Lundberg A. 1957b. Synaptic actions on motoneurones in relation to the two components of the group I muscle afferent volley. J. Physiol 136(3):527–46 13429518 10.1113/jphysiol.1957.sp005778 PMC1358872

[R44] Eccles JC, Schmidt R, Willis WD. 1963. Pharmacological studies on presynaptic inhibition. J. Physiol 168(3):500–30 14067941 10.1113/jphysiol.1963.sp007205 PMC1359437

[R45] Edgley SA. 2001. Organisation of inputs to spinal interneurone populations. J. Physiol 533(Pt. 1):51–56 11351012 10.1111/j.1469-7793.2001.0051b.x PMC2278602

[R46] Edgley SA, Jankowska E. 1987. An interneuronal relay for group I and II muscle afferents in the midlumbar segments of the cat spinal cord. J. Physiol 389(1):647–74 3681739 10.1113/jphysiol.1987.sp016676 PMC1192100

[R47] Edgley SA, Wallace NA. 1989. A short-latency crossed pathway from cutaneous afferents to rat hindlimb motoneurones. J. Physiol 411(1):469–80 2614729 10.1113/jphysiol.1989.sp017584 PMC1190535

[R48] Elson RC. 1987. Flight motor neurone reflexes driven by strain-sensitive wing mechanoreceptors in the locust. J. Comp. Physiol 161(5):747–60

[R49] Engberg I 1964. Reflexes to foot muscles in the cat. Acta Physiol. Scand. Suppl 235:1–64 14188019

[R50] Feldman AG, Orlovsky GN. 1975. Activity of interneurons mediating reciprocal 1a inhibition during locomotion. Brain Res. 84(2):181–94 1111829 10.1016/0006-8993(75)90974-9

[R51] Fink AJP, Croce KR, Huang ZJ, Abbott LF, Jessell TM, Azim E. 2014. Presynaptic inhibition of spinal sensory feedback ensures smooth movement. Nature 509(7498):43–48 24784215 10.1038/nature13276 PMC4292914

[R52] Forssberg H 1979. Stumbling corrective reaction: a phase-dependent compensatory reaction during locomotion. J. Neurophysiol 42(4):936–53 479924 10.1152/jn.1979.42.4.936

[R53] Forssberg H, Grillner S, Rossignol S. 1975. Phase dependent reflex reversal during walking in chronic spinal cats. Brain Res. 85(1):103–7 1109686 10.1016/0006-8993(75)91013-6

[R54] Frye MA, Dickinson MH. 2004. Closing the loop between neurobiology and flight behavior in *Drosophila*. Curr. Opin. Neurobiol 14(6):729–36 15582376 10.1016/j.conb.2004.10.004

[R55] Gatto G, Bourane S, Ren X, Di Costanzo S, Fenton PK, 2021. A functional topographic map for spinal sensorimotor reflexes. Neuron 109(1):91–104 33181065 10.1016/j.neuron.2020.10.003 PMC7790959

[R56] Gaudry Q, Kristan WB Jr. 2009. Behavioral choice by presynaptic inhibition of tactile sensory terminals. Nat. Neurosci 12(11):1450–57 19801989 10.1038/nn.2400

[R57] Gebehart C, Büschges A. 2024. The processing of proprioceptive signals in distributed networks: insights from insect motor control. J. Exp. Biol 227(1):jeb246182 38180228 10.1242/jeb.246182

[R58] Gebehart C, Schmidt J, Büschges A. 2021. Distributed processing of load and movement feedback in the premotor network controlling an insect leg joint. J. Neurophysiol 125(5):1800–13 33788591 10.1152/jn.00090.2021

[R59] Gettrup E 1965. Sensory mechanisms in locomotion: the campaniform sensilla of the insect wing and their function during flight. Cold Spring Harb. Symp. Quant. Biol 30:615–22 5219508 10.1101/sqb.1965.030.01.059

[R60] Gordon S, Dickinson MH. 2006. Role of calcium in the regulation of mechanical power in insect flight. PNAS 103(11):4311–15 16537527 10.1073/pnas.0510109103 PMC1449689

[R61] Gray EG. 1962. A morphological basis for pre-synaptic inhibition? Nature 193(4810):82–83 13901298 10.1038/193082a0

[R62] Grillner S 2006. Biological pattern generation: the cellular and computational logic of networks in motion. Neuron 52(5):751–66 17145498 10.1016/j.neuron.2006.11.008

[R63] Grillner S, Rossignol S. 1978. On the initiation of the swing phase of locomotion in chronic spinal cats. Brain Res. 146(2):269–77 274169 10.1016/0006-8993(78)90973-3

[R64] Grillner S, Williams T, Lagerback PA 1984. The edge cell, possible intraspinal mechanoreceptor. Science 223:500–3 6691161 10.1126/science.6691161

[R65] Gross MK, Dottori M, Goulding M. 2002. Lbx1 specifies somatosensory association interneurons in the dorsal spinal cord. Neuron 34(4):535–49 12062038 10.1016/s0896-6273(02)00690-6

[R66] Hagbarth KE. 1952. Excitatory and inhibitory skin areas for flexor and extensor motoneurons. Acta Physiol. Scand. Suppl 26(94):1–58 14943559

[R67] Hari K, Lucas-Osma AM, Metz K, Lin S, 2022. GABA facilitates spike propagation through branch points of sensory axons in the spinal cord. Nat. Neurosci 25:1288–99 36163283 10.1038/s41593-022-01162-x PMC10042549

[R68] Häring M, Zeisel A, Hochgerner H, Rinwa P, Jakobsson JET, 2018. Neuronal atlas of the dorsal horn defines its architecture and links sensory input to transcriptional cell types. Nat. Neurosci 21(6):869–80 29686262 10.1038/s41593-018-0141-1

[R69] Harris CM, Szczecinski NS, Büschges A, Zill SN. 2022. Sensory signals of unloading in insects are tuned to distinguish leg slipping from load variations in gait: experimental and modeling studies. J. Neurophysiol 128(4):790–807 36043841 10.1152/jn.00285.2022 PMC9529259

[R70] Hasan Z, Stuart DG. 1988. Animal solutions to problems of movement control: the role of proprioceptors. Annu. Rev. Neurosci 11:199–223 3284440 10.1146/annurev.ne.11.030188.001215

[R71] Heide G 1979. Proprioceptive feedback dominates the central oscillator in the patterning of the flight motoneuron output in *Tipula* (Diptera). J. Comp. Physiol 134(2):177–89

[R72] Hess D, Büschges A. 1999. Role of proprioceptive signals from an insect femur-tibia joint in patterning motoneuronal activity of an adjacent leg joint. J. Neurophysiol 81(4):1856–65 10200220 10.1152/jn.1999.81.4.1856

[R73] Hiebert GW, Whelan PJ, Prochazka A, Pearson KG. 1996. Contribution of hind limb flexor muscle afferents to the timing of phase transitions in the cat step cycle. J. Neurophysiol 75(3):1126–37 8867123 10.1152/jn.1996.75.3.1126

[R74] Hofmann T, Koch UT, Bässler U. 1985. Physiology of the femoral chordotonal organ in the stick insect, *Cuniculina impigra*. J. Exp. Biol 114(1):207–23

[R75] Houk J, Henneman E. 1967. Responses of Golgi tendon organs to active contractions of the soleus muscle of the cat. J. Neurophysiol 30(3):466–81 6037588 10.1152/jn.1967.30.3.466

[R76] Hultborn H, Jankowska E, Lindstrom S. 1971. Recurrent inhibition of interneurones monosynaptically activated from group Ia afferents. J. Physiol 215(3):613–36 4253675 10.1113/jphysiol.1971.sp009488 PMC1331904

[R77] Jankowska E 1992. Interneuronal relay in spinal pathways from proprioceptors. Prog. Neurobiol 38(4):335–78 1315446 10.1016/0301-0082(92)90024-9

[R78] Jankowska E 2001. Spinal interneuronal systems: identification, multifunctional character and reconfiguration in mammals. J. Physiol 533(13):31–40 11351010 10.1111/j.1469-7793.2001.0031b.x PMC2278593

[R79] Jankowska E 2022. Basic principles of processing of afferent information by spinal interneurons. J. Neurophysiol 128(3):689–95 36043802 10.1152/jn.00344.2022

[R80] Jankowska E, McCrea DA. 1983. Shared reflex pathways from Ib tendon organ afferents and Ia muscle spindle afferents in the cat. J. Physiol 338(1):99–111 6224005 10.1113/jphysiol.1983.sp014663 PMC1197184

[R81] Kardon AP, Polgár E, Hachisuka J, Snyder LM, Cameron D, 2014. Dynorphin acts as a neuromodulator to inhibit itch in the dorsal horn of the spinal cord. Neuron 82(3):573–86 24726382 10.1016/j.neuron.2014.02.046 PMC4022838

[R82] Kendall MD. 1970. The anatomy of the tarsi of *Schistocerca gregaria* Forskål. Z. Zellforsch. Mikrosk. Anat 109(1):112–37 4918626 10.1007/BF00364935

[R83] Kiehn O 2016. Decoding the organization of spinal circuits that control locomotion. Nat. Rev. Neurosci 17(4):224–38 26935168 10.1038/nrn.2016.9 PMC4844028

[R84] Kittmann R, Schmitz J, Büschges A. 1996. Premotor interneurons in generation of adaptive leg reflexes and voluntary movements in stick insects. J. Neurobiol 31(4):512–32 8951108 10.1002/(SICI)1097-4695(199612)31:4<512::AID-NEU10>3.0.CO;2-F

[R85] Koch SC, Acton D, Goulding M. 2018. Spinal circuits for touch, pain, and itch. Annu. Rev. Physiol 80:189–217 28961064 10.1146/annurev-physiol-022516-034303 PMC5891508

[R86] Koch SC, Del Barrio MG, Dalet A, Gatto G, Günther T, 2017. RORβ spinal interneurons gate sensory transmission during locomotion to secure a fluid walking gait. Neuron 96(6):1419–31.e5 29224725 10.1016/j.neuron.2017.11.011 PMC5828033

[R87] Laurent G, Hustert R. 1988. Motor neuronal receptive fields delimit patterns of motor activity during locomotion of the locust. J. Neurosci 8(11):4349–66 2846797 10.1523/JNEUROSCI.08-11-04349.1988 PMC6569495

[R88] Lesser E, Azevedo AW, Phelps JS, Elabbady L, Cook A, 2024. Synaptic architecture of leg and wing premotor control networks in *Drosophila*. Nature 631(8020):369–77 38926579 10.1038/s41586-024-07600-z PMC11356479

[R89] Levine AJ, Hinckley CA, Hilde KL, Driscoll SP, Poon TH, 2014. Identification of a cellular node for motor control pathways. Nat. Neurosci 17(4):586–93 24609464 10.1038/nn.3675 PMC4569558

[R90] Li J, Zhang W, Guo Z, Wu S, Jan LY, Jan Y-N. 2016. A defensive kicking behavior in response to mechanical stimuli mediated by *Drosophila* wing margin bristles. J. Neurosci 36(44):11275–82 27807168 10.1523/JNEUROSCI.1416-16.2016 PMC5148243

[R91] Li L, Rutlin M, Abraira VE, Cassidy C, Kus L, 2011. The functional organization of cutaneous low-threshold mechanosensory neurons. Cell 147(7):1615–27 22196735 10.1016/j.cell.2011.11.027 PMC3262167

[R92] Lin S, Hari K, Black S, Khatmi A, Fouad K, 2023. Locomotor-related propriospinal V3 neurons produce primary afferent depolarization and modulate sensory transmission to motoneurons. J. Neurophysiol 130(4):799–823 37609680 10.1152/jn.00482.2022 PMC10650670

[R93] Mantziaris C, Bockemühl T, Büschges A. 2020. Central pattern generating networks in insect locomotion. Dev. Neurobiol 80(1–2):16–30 32128970 10.1002/dneu.22738

[R94] Marshall KL, Chadha M, deSouza LA, Sterbing-D’Angelo SJ, Moss CF, Lumpkin EA. 2015. Somatosensory substrates of flight control in bats. Cell Rep. 11(6):851–58 25937277 10.1016/j.celrep.2015.04.001 PMC4643944

[R95] Matthews PBC. 2015. Where Anatomy led, Physiology followed: a survey of our developing understanding of the muscle spindle, what it does and how it works. J. Anat 227(2):104–14 26179022 10.1111/joa.12345 PMC4523315

[R96] Maxwell DJ, Riddell JS. 1999. Axoaxonic synapses on terminals of group II muscle spindle afferent axons in the spinal cord of the cat: axoaxonic synapses on muscle afferent terminals. Eur. J. Neurosci 11(6):2151–59 10336683 10.1046/j.1460-9568.1999.00632.x

[R97] McCrea DA. 2001. Spinal circuitry of sensorimotor control of locomotion. J. Physiol 533(Pt. 1):41–50 11351011 10.1111/j.1469-7793.2001.0041b.x PMC2278617

[R98] Medeiros AM, Hobbiss AF, Borges G, Moita M, Mendes CS. 2024. Mechanosensory bristles mediate avoidance behavior by triggering sustained local motor activity in *Drosophila melanogaster*. Curr. Biol 34(13):2812–30.e5 38861987 10.1016/j.cub.2024.05.021

[R99] Müller T, Brohmann H, Pierani A, Heppenstall PA, Lewin GR, 2002. The homeodomain factor Lbx1 distinguishes two major programs of neuronal differentiation in the dorsal spinal cord. Neuron 34(4):551–62 12062039 10.1016/s0896-6273(02)00689-x

[R100] Oliver KM, Florez-Paz DM, Badea TC, Mentis GZ, Menon V, de Nooij JC. 2021. Molecular correlates of muscle spindle and Golgi tendon organ afferents. Nat. Commun 12(1):1451 33649316 10.1038/s41467-021-21880-3 PMC7977083

[R101] Pabst H 1965. Elektrophysiologische Untersuchung des Streckrezeptors am Flügelgelenk der Wander-heuschrecke *Locusta migratoria*. Z. Vgl. Physiol 50:498–541

[R102] Pearson KG. 2008. Role of sensory feedback in the control of stance duration in walking cats. Brain Res. Rev 57(1):222–27 17761295 10.1016/j.brainresrev.2007.06.014

[R103] Pearson KG, Collins DF. 1993. Reversal of the influence of group Ib afferents from plantaris on activity in medial gastrocnemius muscle during locomotor activity. J. Neurophysiol 70(3):1009–17 8229157 10.1152/jn.1993.70.3.1009

[R104] Pearson KG, Ramirez JM. 1990. Influence of input from the forewing stretch receptors on motoneurones in flying locusts. J. Exp. Biol 151(1):317–40

[R105] Pearson KG, Wong RK, Fourtner CR. 1976. Connexions between hair-plate afferents and motoneurones in the cockroach leg. J. Exp. Biol 64(1):251–66 5571 10.1242/jeb.64.1.251

[R106] Perry SD, McIlroy WE, Maki BE. 2000. The role of plantar cutaneous mechanoreceptors in the control of compensatory stepping reactions evoked by unpredictable, multi-directional perturbation. Brain Res. 877(2):401–6 10986360 10.1016/s0006-8993(00)02712-8

[R107] Picton LD, Bertuzzi M, Pallucchi I, Fontanel P, Dahlberg E, 2021. A spinal organ of proprioception for integrated motor action feedback. Neuron 109(7):1188–201.e7 33577748 10.1016/j.neuron.2021.01.018

[R108] Poulet JFA, Hedwig B. 2002. A corollary discharge maintains auditory sensitivity during sound production. Nature 418(6900):872–76 12192409 10.1038/nature00919

[R109] Prätorius B, Kimmeskamp S, Milani TL. 2003. The sensitivity of the sole of the foot in patients with Morbus Parkinson. Neurosci. Lett 346(3):173–76 12853112 10.1016/s0304-3940(03)00582-2

[R110] Prochazka A, Gorassini M. 1998. Ensemble firing of muscle afferents recorded during normal locomotion in cats. J. Physiol 507(Pt. 1):293–304 9490855 10.1111/j.1469-7793.1998.293bu.x PMC2230769

[R111] Proske U, Gandevia SC. 2012. The proprioceptive senses: their roles in signaling body shape, body position and movement, and muscle force. Physiol. Rev 92(4):1651–97 23073629 10.1152/physrev.00048.2011

[R112] Ramirez JM, Pearson KG. 1988. Generation of motor patterns for walking and flight in motoneurons supplying bifunctional muscles in the locust. J. Neurobiol 19(3):257–82 3373206 10.1002/neu.480190307

[R113] Rossignol S, Dubuc R, Gossard J-P. 2006. Dynamic sensorimotor interactions in locomotion. Physiol. Rev 86(1):89–154 16371596 10.1152/physrev.00028.2005

[R114] Rudomin P, Schmidt RF. 1999. Presynaptic inhibition in the vertebrate spinal cord revisited. Exp. Brain Res 129(1):1–37 10550500 10.1007/s002210050933

[R115] Sathyamurthy A, Johnson KR, Matson KJE, Dobrott CI, Li L, 2018. Massively parallel single nucleus transcriptional profiling defines spinal cord neurons and their activity during behavior. Cell Rep. 22(8):2216–25 29466745 10.1016/j.celrep.2018.02.003 PMC5849084

[R116] Sauer AE, Büschges A, Stein W. 1997. Role of presynaptic inputs to proprioceptive afferents in tuning sensorimotor pathways of an insect joint control network. J. Neurobiol 32(4):359–76 9087889 10.1002/(sici)1097-4695(199704)32:4<359::aid-neu1>3.0.co;2-5

[R117] Sauer AE, Driesang RB, Büschges A, Bässler U. 1996. Distributed processing on the basis of parallel and antagonistic pathways simulation of the femur-tibia control system in the stick insect. J. Comput. Neurosci 3(3):179–98 8872700 10.1007/BF00161131

[R118] Schouenborg J 2002. Modular organisation and spinal somatosensory imprinting. Brain Res. Rev 40(1–3):80–91 12589908 10.1016/s0165-0173(02)00191-1

[R119] Seki K, Perlmutter SI, Fetz EE. 2003. Sensory input to primate spinal cord is presynaptically inhibited during voluntary movement. Nat. Neurosci 6(12):1309–16 14625555 10.1038/nn1154

[R120] Sherrington CS. 1906. The Integrative Action of the Nervous System. New York: Charles Scribner’s Sons

[R121] Siegler MV, Burrows M. 1986. Receptive fields of motor neurons underlying local tactile reflexes in the locust. J. Neurosci 6(2):507–13 3950708 10.1523/JNEUROSCI.06-02-00507.1986 PMC6568518

[R122] Solodkin M, Jiménez I, Rudomin P. 1984. Identification of common interneurons mediating pre- and postsynaptic inhibition in the cat spinal cord. Science 224(4656):1453–56 6328657 10.1126/science.6328657

[R123] Stein W, Schmitz J. 1999. Multimodal convergence of presynaptic afferent inhibition in insect proprioceptors. J. Neurophysiol 82(1):512–14 10400981 10.1152/jn.1999.82.1.512

[R124] Sterbing-D’Angelo S, Chadha M, Chiu C, Falk B, Xian W, 2011. Bat wing sensors support flight control. PNAS 108(27):11291–96 21690408 10.1073/pnas.1018740108 PMC3131348

[R125] Stevenson PA. 1996. Reflex activation of locust flight motoneurones by proprioceptors responsive to muscle contractions. J. Comp. Physiol 180(1):91–98

[R126] Takeoka A, Vollenweider I, Courtine G, Arber S. 2014. Muscle spindle feedback directs locomotor recovery and circuit reorganization after spinal cord injury. Cell 159(7):1626–39 25525880 10.1016/j.cell.2014.11.019

[R127] Taylor A, Gottlieb S. 1985. Convergence of several sensory modalities in motor control. In Feedback and Motor Control in Invertebrates and Vertebrates, ed. Barnes WJP, Gladden MH, pp. 77–91. Dordrecht, Neth.: Springer

[R128] Tuthill JC, Azim E. 2018. Proprioception. Curr. Biol 28(5):R194–203 29510103 10.1016/j.cub.2018.01.064

[R129] Verbe A, Lea KM, Fox JL, Dickerson BH. 2024. Flies tune the activity of their multifunctional gyroscope. Curr. Biol 34(16):3644–53.e3 39053466 10.1016/j.cub.2024.06.066 PMC11338719

[R130] Watson AHD. 1992. Presynaptic modulation of sensory afferents in the invertebrate and vertebrate nervous system. Comp. Biochem. Physiol. Comp. Physiol 103(2):227–39 1359948 10.1016/0300-9629(92)90573-9

[R131] Wendler G 1978. The possible role of fast wing reflexes in locust flight. Sci. Nat 65(1):65–66

[R132] Whelan PJ, Hiebert GW, Pearson KG. 1995. Stimulation of the group I extensor afferents prolongs the stance phase in walking cats. Exp. Brain Res 103(1):20–30 7615034 10.1007/BF00241961

[R133] Windhorst U 2007. Muscle proprioceptive feedback and spinal networks. Brain Res. Bull 73(4–6):155–202 17562384 10.1016/j.brainresbull.2007.03.010

[R134] Witney AG, Wing A, Thonnard J-L, Smith AM. 2004. The cutaneous contribution to adaptive precision grip. Trends Neurosci. 27(10):637–43 15374677 10.1016/j.tins.2004.08.006

[R135] Wolf H 1992. Reflex modulation in locusts walking on a treadwheel intracellular recordings from motoneurons. J. Comp. Physiol 170(4):443–62

[R136] Wolf H, Burrows M. 1995. Proprioceptive sensory neurons of a locust leg receive rhythmic presynpatic inhibition during walking. J. Neurosci 15(8):5623–36 7643206 10.1523/JNEUROSCI.15-08-05623.1995 PMC6577635

[R137] Wolf H, Büschges A. 1995. Nonspiking local interneurons in insect leg motor control. II. Role of nonspiking local interneurons in the control of leg swing during walking. J. Neurophysiol 73(5):1861–75 7623086 10.1152/jn.1995.73.5.1861

[R138] Wolf H, Pearson KG. 1988. Proprioceptive input patterns elevator activity in the locust flight system. J. Neurophysiol 59(6):1831–53 3404207 10.1152/jn.1988.59.6.1831

[R139] Woo S-H, Lukacs V, de Nooij JC, Zaytseva D, Criddle CR, 2015. Piezo2 is the principal mechanotransduction channel for proprioception. Nat. Neurosci 18(12):1756–62 26551544 10.1038/nn.4162 PMC4661126

[R140] Wu H, Petitpré C, Fontanet P, Sharma A, Bellardita C, 2021. Distinct subtypes of proprioceptive dorsal root ganglion neurons regulate adaptive proprioception in mice. Nat. Commun 12(1):1026 33589589 10.1038/s41467-021-21173-9 PMC7884389

[R141] Wyart C, Carbo-Tano M, Cantaut-Belarif Y, Orts-Del’Immagine A, Böhm UL. 2023. Cerebrospinal fluid-contacting neurons: multimodal cells with diverse roles in the CNS. Nat. Rev. Neurosci 24(9):540–56 37558908 10.1038/s41583-023-00723-8

[R142] Zehr EP, Stein RB, Komiyama T. 1998. Function of sural nerve reflexes during human walking. J. Physiol 507(Pt. 1):305–14 9490858 10.1111/j.1469-7793.1998.305bu.x PMC2230764

[R143] Zhang J, Lanuza GM, Britz O, Wang Z, Siembab VC, 2014. V1 and V2b interneurons secure the alternating flexor-extensor motor activity mice require for limbed locomotion. Neuron 82(1):138–50 24698273 10.1016/j.neuron.2014.02.013 PMC4096991

[R144] Zimmerman AL, Kovatsis EM, Pozsgai RY, Tasnim A, Zhang Q, Ginty DD. 2019. Distinct modes of presynaptic inhibition of cutaneous afferents and their functions in behavior. Neuron 102(2):420–34.e8 30826183 10.1016/j.neuron.2019.02.002 PMC6472967

